# An Ethical Reflection on Assisted Dying, Inequity Within Palliative Medicine Services and Access to Care in the United Kingdom: A Resident Doctor's Perspective

**DOI:** 10.7759/cureus.96578

**Published:** 2025-11-11

**Authors:** Morgan W Hayden

**Affiliations:** 1 Internal Medicine, The Grange University Hospital, Cwmbran, GBR

**Keywords:** active and passive euthanasia, assisted dying, end of life and hospice care, medical ethics, palliative medicine research

## Abstract

With the passing of the Assisting Dying Act in the United Kingdom, there are growing concerns from within and outside the Palliative Care community. The conflicting ethical issues at play are complex and often contradictory. I present a short summary of several ethical issues and a narrative argument in opposition to assisted dying in our current system, citing autonomy, beneficence, arguments of justice, inequity and a rights-based approach. Overall, I will argue that the disparity between availability and demand for access to specialist palliative care creates an inequitable playing field for patients and clinicians and that until equitable access to palliative care is established, patients are at risk of being consented for assisted dying without access to a viable and evidence-based alternative.

## Editorial

Assisted dying, generally defined as the ‘ending of one’s life with the assistance of another’, is contrasted with euthanasia only in so much that in the former the individual administers the drugs themselves, in euthanasia an external agent administers the medication. For the purposes of this discussion, I will consider them broadly autologous [1]. Indeed, from a narrative perspective, there does appear to be little difference between prescribing a medication intending it to be administered and the act of administering it. The intention to kill is identical in both scenarios, and this is broadly supported in philosophical literature [2]. 

Beneficence [3] can be argued both as a proponent and antagonist to assisted dying. For the proponents of assisted dying, the argument would follow that the state of non-existence yielded by death is greater than that of the suffering, perceived or otherwise, of being alive [4]. Counter to this, opponents would argue that it cannot be argued that the state of non-existence is preferable, in that it inherently destroys the agent and hence how can one’s wellbeing have been said to have improved by not existing?

Touching on a rights-based approach to assisted dying, in current UK law, generally speaking, an individual does not have the right to demand intervention or treatment, only to refuse treatment offered. It would be nonsensical and unethical to give treatment that would only cause harm. It is well established that the withdrawal of life-sustaining treatment is legal in the UK, insomuch as it allows the physiological process of disease to proceed uninterrupted. Whilst it may be argued that assisted dying creates a more equitable playing field for autonomous decisions about death, the person with a longstanding and painful neurological condition may now die on a similar timeline as the individual with pneumonia; it fails to recognise that clinicians are not agents in the mechanism of death. Nor does it recognise that fundamentally, assisted dying is antagonistic to the tenets of Modern Medical practice, causing death does not promote health, it is not healthcare, and is in opposition to the principle of non-maleficence, a concept that can be summarised as not causing or perpetrating harm [3]. Furthermore, in Western society, for example, the early death of an individual is seen as a negative outcome regardless of the degree of suffering. Such that those who wish to bring harm to themselves are restrained and protected for their own safety through various mechanisms. Is there something intrinsically less valuable about the life of an individual with a life-shortening illness than without?

Autonomy, self-rule, plausibly supports the right to self-determination and the patient’s wish to die. The degree to which individuals are autonomous in making this decision is less clear [3]. Proponents of assisted dying would argue that for those in ‘unbearable suffering’, the choice to die is an inalienable right. However, whilst maintaining an individual’s ‘first order autonomy’, we may undermine ‘second-order autonomy’ in the process. Second-order autonomy describes the higher and arguably truer desires of an individual. For example, an individual may choose to eat a chocolate bar in keeping with their first-order autonomy, but denying them this may align with their second-order autonomy, to stay on their diet. Fundamentally, the essence of this argument rests on an individual's ability to make an informed decision, and this touches on arguments of justice and equity. 

The argument may follow, ‘20 people in the UK die every day in pain, we should respect their right to self-rule and allow them to die without pain in keeping with their wishes' [5]. What this fails to establish is the individuals’ true wishes; they may wish to be pain free, to live with dignity, but perceive their current state now to be unlivable, without any alternatives. 

There are two prima facie issues with this: firstly, we do not know what this individual’s perspective will be in one day, one week, or one year. Nor their wishes, if they receive adequate, evidence-based Palliative Medicine, in a system with consistent and reliable social care, which I will discuss below. Does the hypothetical risk of ongoing suffering outweigh the state of non-existence? To put it bluntly, this is a decision that is irrevocable, unlike many medical interventions, hence the threshold for alternative approaches must be higher. 

Secondly, applying this to the earlier example of first- and second-order autonomy, by offering an individual assisted dying, we are plausibly giving them an autonomous and free decision, they chose to die, and hence are rid of their pain. However, their desire may not be to die, but they consider their current situation undignified or too painful and cannot conceive an alternative. These higher order desires could be addressed by timely access to specialist services. Hence, whilst we may be promoting choice, and plausibly therefore respecting autonomy, what really matters is the environment in which this decision is made. 

This is troubling because we know there is a well-documented inequity within the United Kingdom for access to and funding of specialist Palliative Medicine. For example, 27% of areas in the UK offer no designated support at night or weekends; moreover, only 30% offer telephone support [6]. Additionally, whilst much of the care delivered in the UK is nationally funded, the majority of specialist hospice care is supported or indeed run by charitable organisations. Indeed, only 30% of funding for hospice care is provided by the NHS [7]. Moreover, this is reflected in our patient’s perspective of Palliative Medicine; for example, a Kings College London sponsored YouGov survey found that 65% of adults are worried about access to Palliative Medicine [8]. We know access to palliative medicine and specialist palliative medicine services improves pain management [9], perceptions of dignity and anxiety [10], and is fundamental in improving quality of life. If the perception is that this service is simply not available, assisted dying may look like a plausible, if not the only, alternative. 

Furthermore, this problem will only compound; the need for palliative medicine is expected to increase, with demand posited to rise by 42% by 2040 [11]. The ethical arguments in favour of assisted dying seldom take into account this growing inequity within the speciality, indeed I would argue that the very fact that 20 people a day die in the UK in pain, is not an argument for assisted dying but for more reliable, consistent and specialist Palliative Medicine and social care provision [5]. With better access to equitable, timely, and evidence-based medicine, a number of those who would have chosen assisted dying may not do so, and those who do choose to proceed would be able to do so in a truly autonomous fashion. Hence, until this is achieved, the delivery of assisted dying is likely to do a disservice to some of our most deserving patients. 

Overall, while there may be permissible ethical and moral arguments in favour of assisted dying, including those of beneficence and autonomy, one cannot help but feel uncomfortable with the scale of inequity within services and access to specialist care. Within this framework, it is hard to see how individuals are in a position to make truly informed and autonomous decisions about the nature and time of their death. Applying the principle of non-maleficence, it is clear that clinicians and the services they work in have a duty to offer viable alternatives to assisted dying in the first instance. At present, this is simply not the case; it therefore follows that the prima facie tenet of medical practice ‘primum non nocere’, first do no harm, is in conflict, albeit with best intentions of the proponents. Whilst this conflict is common and rife in medical practice, for example, the pain of the needle breaking skin, conflicting with the benefit of the blood test, never before have the stakes, and indeed the risks of harm, been higher. 

With this, the most important decision people can make, it is imperative that individuals have access to all available options, including access to timely, sustainable, and reliable specialist palliative medicine. Until this is met, with well-funded, robust and equitable access for patients, offering assisted dying presents a false and arguably unethical choice to our patients. 
